# The RST and PARP-like domain containing SRO protein family: analysis of protein structure, function and conservation in land plants

**DOI:** 10.1186/1471-2164-11-170

**Published:** 2010-03-12

**Authors:** Pinja Jaspers, Kirk Overmyer, Michael Wrzaczek, Julia P Vainonen, Tiina Blomster, Jarkko Salojärvi, Ramesha A Reddy, Jaakko Kangasjärvi

**Affiliations:** 1 Plant Biology Division, Department of Biosciences, University of Helsinki, Viikinkaari 1, FI-00014 Helsinki, Finland

## Abstract

**Background:**

The SROs (SIMILAR TO RCD-ONE) are a group of plant-specific proteins which have important functions in stress adaptation and development. They contain the catalytic core of the poly(ADP-ribose) polymerase (PARP) domain and a C-terminal RST (RCD-SRO-TAF4) domain. In addition to these domains, several, but not all, SROs contain an N-terminal WWE domain.

**Results:**

SROs are present in all analyzed land plants and sequence analysis differentiates between two structurally distinct groups; cryptogams and monocots possess only group I SROs whereas eudicots also contain group II. Group I SROs possess an N-terminal WWE domain (PS50918) but the WWE domain is lacking in group II SROs. Group I domain structure is widely represented in organisms as distant as humans (for example, HsPARP11). We propose a unified nomenclature for the SRO family. The SROs are able to interact with transcription factors through the C-terminal RST domain but themselves are generally not regulated at the transcriptional level. The most conserved feature of the SROs is the catalytic core of the poly(ADP-ribose) polymerase (PS51059) domain. However, bioinformatic analysis of the SRO PARP domain fold-structure and biochemical assays of AtRCD1 suggested that SROs do not possess ADP-ribosyl transferase activity.

**Conclusions:**

The SROs are a highly conserved family of plant specific proteins. Sequence analysis of the RST domain implicates a highly preserved protein structure in that region. This might have implications for functional conservation. We suggest that, despite the presence of the catalytic core of the PARP domain, the SROs do not possess ADP-ribosyl transferase activity. Nevertheless, the function of SROs is critical for plants and might be related to transcription factor regulation and complex formation.

## Background

The RCD1 (RADICAL-INDUCED CELL DEATH1) protein is an important regulator of plant stress and developmental responses [1,2]. In *Arabidopsis thaliana *it is a member of a small protein family consisting of RCD1 and five SROs (SIMILAR-TO-RCD-ONE). *RCD1 *was first identified as a plant gene able to complement the oxidative stress sensitive phenotype of a yeast strain deficient in the YAP1 transcription factor [3]. Since then it has also been characterized as a major regulator of plant ozone (O_3_) tolerance [4]. A loss-of-function mutation in *RCD1 *results in highly pleiotropic phenotypes including increased sensitivity to extracellular reactive oxygen species (ROS), resistance to chloroplastic ROS formation by paraquat (methyl viologen) and ultraviolet radiation, salt sensitivity, aberrant leaf and rosette morphology, early flowering, altered nitric oxide and hormone (jasmonic acid and ethylene) responses, as well as defects in developmental processes, such as root architecture and reproductive development [1,2,4-9]. While *rcd1 *displays a vast range of well-characterized phenotypes, the function of its closest ortholog, *SRO1*, is dispensable for normal plant development and stress response [1]. Mutant *sro1 *plants exhibit only very subtle phenotypes [2]. However, loss of a single *SRO1 *allele in *rcd1 *background results in severe developmental defects with the *rcd1 sro1 *double mutant displaying even more extreme phenotypes [1,2]. This demonstrates unequal genetic redundancy between *RCD1 *and *SRO1 *in *A. thaliana *[1,10]. In species other than *A. thaliana*, several studies, mostly based on gene expression analysis, suggest roles for RCD1 and SRO1 orthologs in hormone signaling, plant development and response to biotic and abiotic stresses [1,2,11-17]. However, the phylogenetic relationships of these proteins to the RCD1/SRO gene family members in *A. thaliana *has so far not been characterized.

Another member of the *A. thaliana *SRO family, *SRO5 *(*At5g62520*), is transcriptionally induced by ROS in response to salt treatment and is required for the proper response to oxidative stress [18]. It forms an antisense overlapping gene pair with Δ^1^-pyrroline-5-carboxylate dehydrogenase (*P5CHD*). In the presence of both transcripts, a 24-nucleotide siRNA is formed, downregulating expression of *P5CDH *[18]. A salt stress responsive *SRO5 *ortholog from tomato can functionally complement the *A. thaliana sro5 *mutant [19]. The other members of the SRO protein family, SRO2, SRO3 and SRO4, have not been functionally analyzed.

The domain composition of the SROs is unique within plants. While two *A. thaliana *SRO family members contain an N-terminal WWE domain (PS50918 [20]), all of them are characterized by the possession of the core of the poly(ADP-ribose) polymerase (PARP; PS51059) domain and a conserved C-terminal RCD1-SRO-TAF4 domain (RST domain; PF12174) [1]. The combination of PARP and RST domain is specific to plants but the WWE-PARP domain architecture is widely conserved in organisms as distantly related as humans. The WWE domain is involved in protein-protein interactions and predicted to have a globular structure [20,21]. However, in SROs the function of the WWE domain in dimerization and other protein-protein interactions remains to be shown. The RST domain is a plant specific domain found in plant WWE-PARPs and TAF4s (TBP-Associated Factor 4), a component of the TFIID general transcription factor. The RST domain-bearing C-termini of RCD1 and SRO1 are suggested to be critical for the interaction with several, mostly plant specific transcription factors [1].

Protein ADP-ribosylation is a post-translational modification catalyzed by ADP-ribosyl transferases (ARTs) that are present in all eukaryotes except yeast [22]. The major classes of ARTs are the PARPs and mono(ADP-ribosyl) transferases (mARTs). PARPs attach single ADP-ribose units to proteins and catalyze the elongation and branching of long poly(ADP-ribose) chains. PARPs have roles in many processes, including cell death, DNA repair, telomere stability, chromatin remodeling, transcription, and memory [23]. *A. thaliana *has three PARPs which most closely resemble classical DNA dependent PARPs http://www.arabidopsis.org/. The presence of the catalytic core of the PARP domain in RCD1 and SROs suggests an ART or related activity.

The mARTs attach a single ADP-ribose unit to protein substrates. Humans possess both ectoenzymes and intracellular endogenous mARTs [24,25]. To date, in plants no mARTs have been isolated or predicted by bioinformatics [26,27]. Most known human intracellular mARTs resemble PARPs [26] and have until recently been classified as PARPs [24]. There are 11 such human PARPs with various domain structures. HsPARP7, HsPARP12, HsPARP13 and HsPARP14 contain the WWE and PARP domain together with other domains. HsPARP11, with only WWE and PARP but no other conserved domains, is the human protein most similar in domain architecture to *A. thaliana *RCD1 and SRO1 and has currently no known function. Given the evolutionary distance between plants and humans it is not clear which, if any, of these proteins are functionally similar to the SROs.

The identification of *RCD1 *orthologs in several plant species prompted us to investigate the SRO protein family in a comparative manner. The availability of sequenced and annotated genomes allows the analysis of full gene families *in silico*. We compared the SRO family in several species from evolutionarily divergent branches of the plant kingdom showing a different composition of the family in different plants. In addition, we suggest a naming convention for the family members. We identified the RST domain as a protein-protein interaction domain, analyzed the predicted function of the PARP domain and studied the transcriptional regulation of the gene family members in *A. thaliana*. Based on our findings we propose that, while SROs contain a highly conserved PARP domain, at least RCD1 does not possess ADP-ribosyl transferase activity. Bioinformatic comparisons suggest this is likely to also apply to several other SROs.

## Results and Discussion

**Figure 1:**
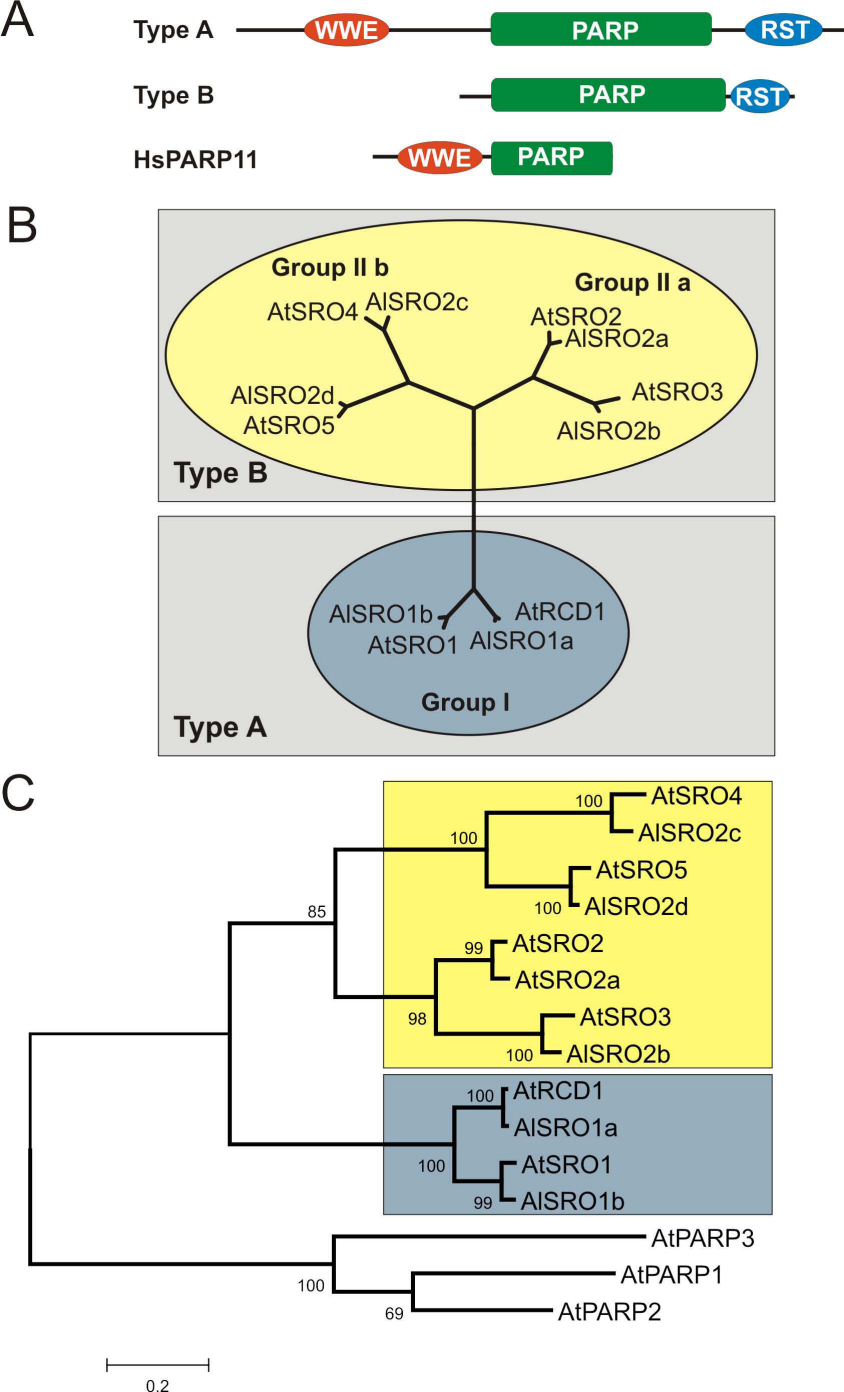
**Structural classes and evolutionary relationships of RCD1 and RCD1-like proteins**. (A) Schematic diagram depicting the protein structure of *A. thaliana *RCD1/SRO protein family members representing the two structural classes, type A (AtRCD1) and B (AtSRO2). RCD1 and all SROs possess a poly(ADP-ribose) polymerase (PARP) catalytic region (PS51059) and a C-terminal RST (RCD1-SRO-TAF4; PF12174) domain. Additionally, the presence (type A) or absence (type B) of a WWE domain (PS50918) differentiates between the two structural classes. Human HsPARP11 exhibits similar domain composition to the *Arabidopsis *SROs containing a WWE domain and the PARP domain. It is representative of the five human WWE-PARPs although the remaining four have additional conserved domains. (B) *A. thaliana *and *A. lyrata *SROs clustered in three groups in an unrooted Neighbour-joining tree. AtRCD1 and AtSRO1 and the *A. lyrata *orthologs AlSRO1a and AlSRO1b formed group I, which structurally belongs to type A. AtSRO2 and AtSRO3 and the *A. lyrata *orthologs AlSRO2a and AlSRO2b formed group IIa. AtSRO4 and AtSRO5 and the *A. lyrata *orthologs AlSRO2c and AlSRO2d form group IIb. All members of group II belong to structural type B. (C) Neighbour-joining tree of the *A. thaliana *and *A. lyrata *SROs rooted the *A. thaliana *PARPs (AtPARP1, 2 and 3). The SRO proteins clustered together and form a monophyletic group while AtPARP1, 2 and 3 clustered together to form a single outgroup for the SRO protein family.

Based on their domain composition the *A. thaliana *SROs could be divided into two structural types (Figure 1A). Type A SROs contain an N-terminal WWE domain (PS50918) [20], the catalytic core of the poly(ADP-ribose) polymerase (PARP; PS51059) domain and a C-terminal RCD1-SRO-TAF4 (RST; PF12174) [1] domain. The type B SROs lack the WWE domain but possess the PARP and RST domains.

The *A. thaliana *SRO protein family consists of six members (Figure 1B), AtRCD1 and AtSRO1 to AtSRO5. Based on a Neighbour-joining tree using full length protein sequences, they formed distinct groups: AtRCD1 and AtSRO1 belong to group I while the others form group II, which is further divided into two subgroups. AtSRO2 and AtSRO3 belong to group IIa and AtSRO4 and AtSRO5 to group IIb. AtRCD1 and AtSRO1 have an identical protein domain structure and belong to structural type A (Figure 1A). HsPARP11 (Figure 1A) and a few other human PARPs possess similar domain structure with an N-terminal WWE domain and a PARP domain as the structural type A SROs, but lack the C-terminal RST domain. Group II SROs (both subgroups) form the structural type B. The closest sequenced relative of *A. thaliana*, *Arabidopsis lyrata*, possesses the same complement of SRO proteins. Orthologs from the two species clustered together in the phylogenetic trees based on the full length protein sequences (Figure 1B) and the PARP domain (Figure 1C).

### Transcriptional regulation of the A. thaliana SROs

**Figure 2:**
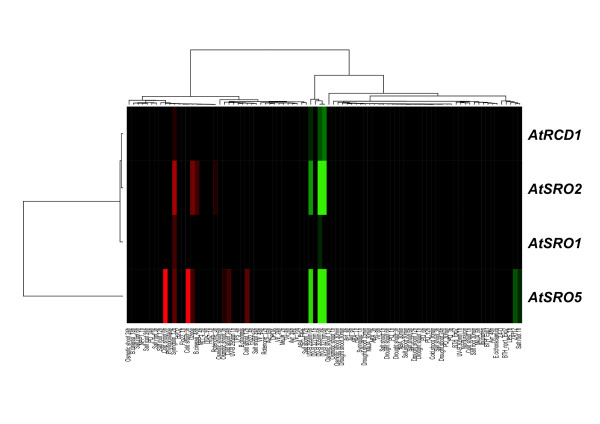
**Transcript profile of SRO family genes**. Bootstrapped Bayesian hierarchical clustering of the *A. thaliana SRO *family genes under various stresses compared to normal growth conditions. The stress data sets were downloaded from public databases (see Methods for complete description of the method and the data). Red and green indicate increased or decreased expression compared to untreated plants, respectively. The intensity of the colors is proportional to the absolute value of the fold difference.

**Figure 3:**
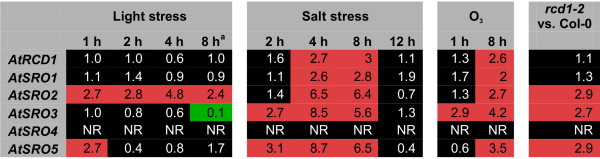
**qPCR analysis of SRO family genes**. Steady state transcript levels of *A. thaliana SRO *family genes were investigated by qPCR. Relative gene expression under light stress, salt stress, and exposure to O_3 _and in the *rcd1-2 *mutant is shown compared to Col-0 wildtype plants grown under normal conditions. Red indicates elevated and green decreased expression. Black indicates unaltered transcript levels or in the case of *AtSRO4 *not reproducible (NR). Numbers indicate relative fold-change ratios. All experiments were repeated three times, one representative experiment is shown.

Three *A. thaliana *SROs, *AtRCD1*, *AtSRO1 *and *AtSRO5 *have previously been functionally characterized. Several studies suggest that the expression of *AtRCD1 *and *AtSRO1 *is developmentally regulated and only slightly stress responsive [1,2,9], whereas *AtSRO5 *has previously been indicated as common stress response gene [28]. To probe transcriptional regulation of the *AtSRO *gene family, we mined publicly available Affymetrix microarray chip data (see Methods; *AtSRO3 *and *AtSRO4 *are not represented on the Affymetrix arrays). These results confirmed that *AtSRO5 *was the transcriptionally most responsive member of the *SRO *family (Figure 2). In order to verify and complement the microarray data, quantitative real-time RT-PCR (qPCR) analyses indicated that *AtRCD1 *and *AtSRO1 *exhibited only subtle regulation in response to stress treatments (Figures 2 and 3). Low variation in transcript abundance in response to stress conditions suggests that these proteins are regulated primarily at the post-translational level under our conditions. This is consistent with the observed low and tightly controlled amounts of RCD1 protein [1]. In contrast to our results, Bechtold *et al*. [16] reported a strong increase in *AtRCD1 *transcript abundance in response to excess light stress. This difference is most likely due to the intensity and quality of the light used. *AtSRO2*, *AtSRO3 *and *AtSRO5 *showed changes in transcript levels in response to light stress, salt treatment and exposure to O_3 _(Figure 3). *AtSRO5 *showed the clearest transcriptional responses to the stress treatments also in the qPCR analysis. No reproducible results were obtained for *AtSRO4 *but its presence in EST databases suggests that it is expressed in plants. Expression of the *SRO *genes was analyzed by qPCR also in the *rcd1-2 *mutant. *SRO2*, 3, and 5 exhibited higher transcript accumulation in *rcd1-2*, suggesting that RCD1 acts as a negative regulator of these other gene family members. The effect could be indirect and due to the *rcd1 *mutant being primed for stress responses [1].

**Figure 4:**
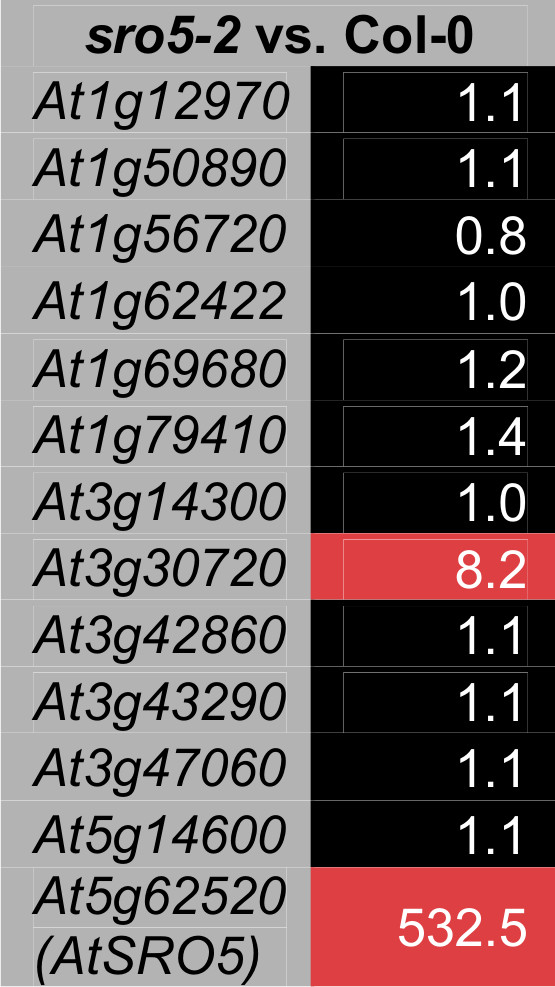
**Real-time quantitative PCR analysis of the sro5-2 mutant**. The expression of 13 genes which were most differentially expressed in non-stressed *sro5-2 *mutant plants according to microarray results (data not shown) was re-examined by qPCR. Red indicates elevated and black unaltered transcript levels compared to Col-0 wildtype plants. Numbers indicate relative fold-change ratios. All experiments were repeated three times, one representative experiment is shown.

The *AtSRO5 *gene forms a natural siRNA pair with its neighbouring gene *P5CDH *in *A. thaliana *where they participate in a regulatory network during ROS-mediated salt responses [18]. Interestingly, in *A. lyrata*, grapevine, or poplar the *P5CDH *gene is not located next to the orthologs of *AtSRO5 *; none of the *AtSRO5 *orthologs overlap with their respective neighbouring genes http://gbrowse.arabidopsis.org/cgi-bin/gbrowse_syn/arabidopsis/. This suggests that the system of *P5CDH *transcript regulation by natural siRNA formation with *SROs *is specific to *A. thaliana*. In order to address *AtSRO5 *gene function in transcriptional regulation, we performed microarray analysis of unstressed *sro5-2 *plants. The *sro5-2 *allele (GABI-325B05) used in our study carries a T-DNA insertion in the second exon and expresses a truncated transcript [19]. Microarray results revealed several genes with altered expression according to the fold-change ratio (data not shown). However, these differences were not supported as significant by statistical analysis. To verify the array results with an independent method, we analyzed the expression of the genes with the clearest fold-changes by qPCR (Figure 4). Similar to Babajani *et al*. [19], *AtSRO5 *itself had increased expression in the *sro5-2 *mutant (Figure 4). Only one other gene, *At3g30720*, encoding *QUA-QUINE-STARCH *[29], exhibited reproducible changes of expression levels in the *sro5-2 *mutant. The expression of *P5CDH*, the *AtSRO5 cis*-antisense gene pair, was not altered according to our results and the study by Babajani *et al*. [19], suggesting that natural siRNA formation might not be the primary regulatory mechanism in unstressed plants despite the elevated *AtSRO5 *transcript.

### SRO conservation and nomenclature in land plants

**Figure 5:**
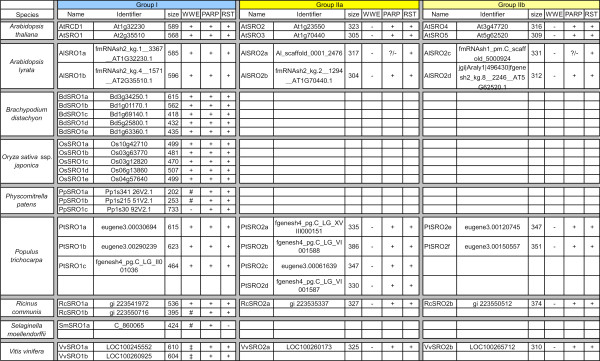
**SRO Orthologs in Sequenced Plant Genomes**. All SRO sequences used for analyses are listed with names according to the proposed nomenclature and their original identifiers. The length of the proteins in amino acids (AAs; size) and the presence (+) or absence (-) of potential conserved domains (WWE PS50918, PARP PS51059, RST PF12174) are indicated. Proteins predicted to lack domains because they are not full length are indicated (#). Domains present but with low statistical support are indicated with (‡). Data source: NCBI (National Center for Bioinformatics, http://www.ncbi.nlm.nih.gov/). Additional web resources are listed below: PZ, Phytozome http://www.phytozome.net/; TAIR, the Arabidopsis Information Resource http://www.arabidopsis.org/; JGI, Joint Genome Initiative (Poplar: http://genome.jgi-psf.org/Poptr1_1/Poptr1_1.home.html; *Physcomitrella*: http://genome.jgi-psf.org/Phypa1_1/Phypa1_1.home.html; *Selaginella*: http://genome.jgi-psf.org/Selmo1/Selmo1.home.html); CVI, Craig Venter Insititute http://castorbean.jcvi.org/; BDB, Brachypodium database http://www.brachypodium.org/; RGADB, Rice Genome Annotation database http://rice.plantbiology.msu.edu/. Two *SROs *from *Brachypodium*, Bradi2g10720.1 and Bradi1g01340.1, were only present as very short and incomplete predictions, and thus could not be assigned to any group.

**Figure 6:**
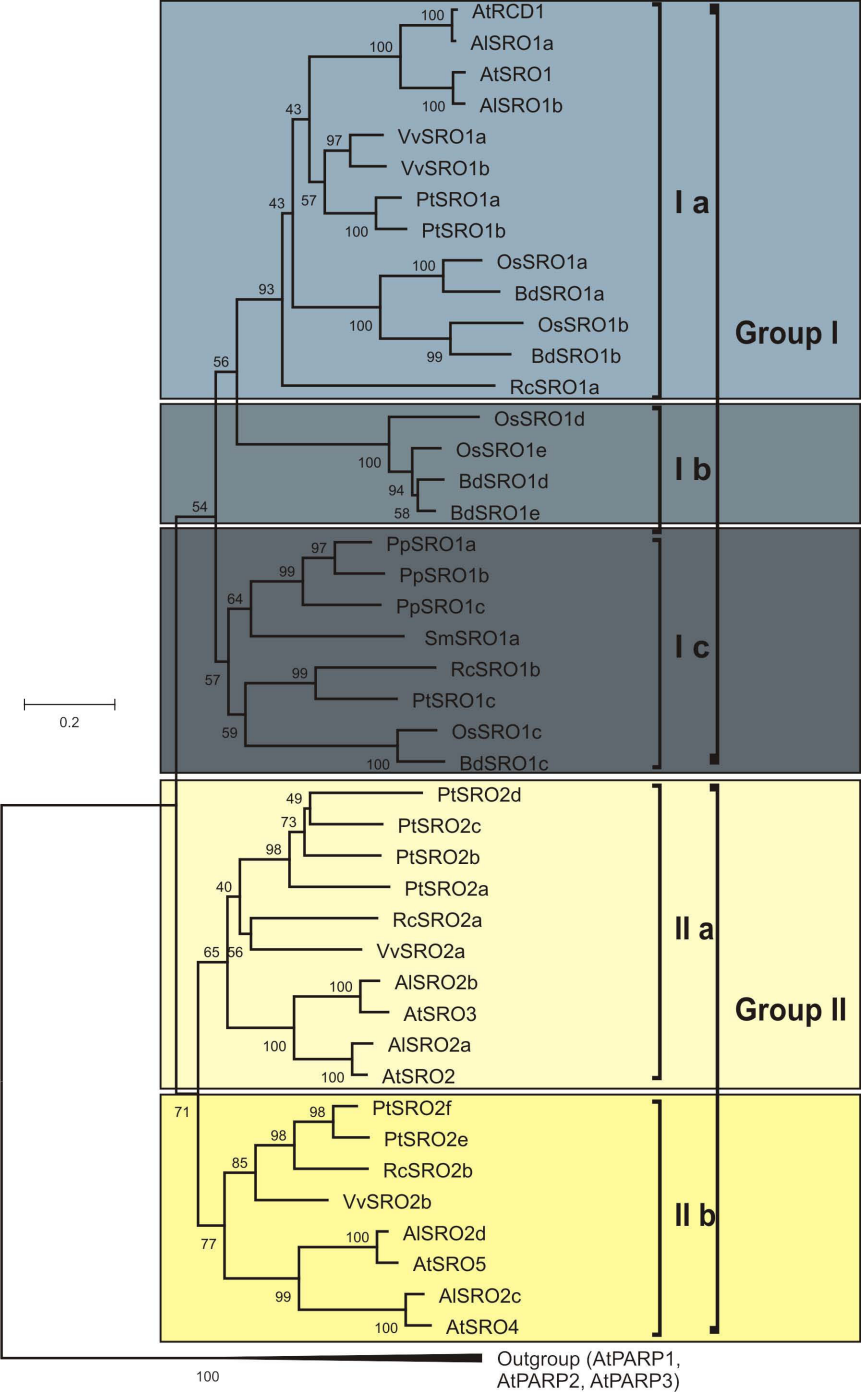
**Neighbour-joining tree of the PARP domains of the plant SRO protein family**. The PARP domains of the SRO proteins from the sequenced genomes of *A. thaliana*, *A. lyrata*, *Vitis vinifera*, *Ricinus communis*, *Populus trichocarpa*, *Oryza sativa *ssp. *japonica*, *Brachypodium distachyon*, *Physcomitrella patens *and *Selaginella moellendorffi *were identified and aligned. Subsequently, a Neighbour-joining phylogenetic tree was constructed using MEGA4. AtPARP1, 2 and 3 were used as outgroups. Plant SROs could be classified into two groups. Group I contained SROs from all included plant species and could be further divided into three subgroups (Ia, Ib and Ic) according to the C-terminal RST domain. Most SROs in group I belonged to structural type A. The members of group II (a and b) without exception belonged to structural type B.

To better understand the structure of the SRO protein family in plants, the *A. thaliana *protein sequences were used to identify and analyze the sequences of SROs in several fully sequenced plant genomes (see Table 1 for list of species names and abbreviations). No SRO protein orthologs were found in the sequenced genomes of algae or photosynthetic bacteria (see Methods). Because no sequence data or EST information is available for any of the streptophyte algae, we cannot exclude the possibility that SROs are present in this group. However, the SRO family was present in all land plant genomes analyzed and showed considerable variation in its composition between plant species (Figures 5 and 6).

**Table 1:** Species of sequenced plant genomes used in this study.

Genomes					
**Species**	**Abbr**.	**Common Name**	**Clade**	**Ref**.	**Data Source**

*Arabidopsis thaliana*	At	Thale cress	Dicot/Eurosid II	[65]	TAIR

*Arabidopsis lyrata*	Al	Lyrate rock cress	Dicot/Eurosid II	-	TAIR

*Brachypodium distachyon*	Bd	Purple false-brome	Monocot/Poales	[66]	BDB

*Oryza sativa *ssp. *japonica*	Os	Rice	Monocot/Poales	[67]	RGADB

*Physcomitrella patens*	Pp	Club moss	Bryophyte	[68]	JGI

*Populus trichocarpa*	Pt	Poplar	Dicot/Eurosid I	[69]	JGI

*Ricinus communis*	Rc	Castor bean	Dicot/Eurosid I	-	CVI

*Selaginella moellendorffi*	Sm	Spikemoss	Lycophyte	-	JGI

*Vitis vinifera*	Vv	Grapevine	Dicot (basal Rosid)	[70]	VGC

List of the species used in this analysis including their abbreviation (abbr.), common name, phylogenetic classification (clade), and sources of data used. Analysis of all species utilized resources of the NCBI: National Center for Bioinformatics http://www.ncbi.nlm.nih.gov/. Additional web resources are listed below according to the abbreviations: PZ, Phytozome http://www.phytozome.net/); TAIR, the Arabidopsis Information Resource http://www.arabidopsis.org/; JGI, Joint Genome Initiative (Poplar: http://genome.jgi-psf.org/Poptr11/Poptr11.home.html; *Physcomitrella*: http://genome.jgi-psf.org/Phypa11/Phypa11.home.html; *Selaginella*: http://genome.jgi-psf.org/Selmo1/Selmo1.home.html); CVI, Craig Venter Insititute http://castorbean.jcvi.org/; BDB, *Brachypodium *database http://www.brachypodium.org/; RGADB, Rice Genome Annotation database http://rice.plantbiology.msu.edu/; VGC (Vitis Genome Consortium), The French-Italian Public Consortium for Grapevine Genome Characterization http://www.cns.fr/spip/vitis-vinifera-e.html.

The lack of clear one-on-one orthology outside the Brassicaceae (see below) rendered naming conventions based on *A. thaliana *impractical for most plant species. Therefore, a new unified nomenclature system is proposed (Figure 5). The key features of this system are: i) *A. thaliana *proteins retain their current names. ii) All RCD1/SRO family members in other species are named SROs and prefixed with a two letter abbreviation of the species scientific name. iii) All SROs are assigned a number designation; i.e. all SRO1s are in group I and SRO2s in group II. iv) Multiple proteins within one group are then assigned an arbitrary letter designation in the order of their discovery to identify them individually. This nomenclature system allows the differentiation between group I and II SROs and will facilitate the comparison of related SROs between species. All proteins used in this study have been named according to these conventions (Figure 5).

### Representation of SRO groups and structural types in land plants

ScanProsite [30,31] and SMART [32,33] were used to identify conserved domains in the SRO protein sequences. The catalytic core of the PARP domain was the most consistently conserved feature of all identified SRO proteins. Therefore, the PARP domains were used for the construction of a phylogenetic Neighbour-joining tree (Figure 6). The tree was rooted using *A. thaliana *classical PARP proteins (AtPARP1, 2 and 3) as an outgroup. AtRCD1/AlSRO1a and also AtSRO1/AlSRO1b from both *Arabidopsis *species grouped tightly and, along with SROs from grapevine, poplar, castor bean, rice, and *Brachypodium distachyon*, formed the subgroup Ia. These proteins are of the structural type A containing WWE, PARP, and RST domains (Figure 1A). The second subgroup Ib contains only proteins from the grasses rice and *B. distachyon*. This subgroup includes only structural type A proteins (Figure 1A). The orthologs from the moss *Physcomitrella patens *and the representative of basal vascular plants *Selaginella moellendorffi*, together with sequences from castor bean, poplar, rice, and *B. distachyon*, formed the subgroup Ic. These proteins retain the PARP and RST domains while the WWE domain is only present in PtSRO1c, OsSRO1c, and BdSRO1c. Of the group Ic members in which no WWE domain was detected, only PpSRO1c appears to be a full-length sequence (Figure 5).

The group IIa (Figure 6) contains AtSRO2 and 3, which grouped with their orthologs from *A. lyrata *(AlSRO2a and b, respectively). The other members of group II are VvSRO2a, RcSRO2a, and a group of 4 closely related orthologs from poplar (PtSRO2a, b, c and d). The group IIb (Figure 6) contains AtSRO4 and 5, which clustered together with AlSRO2c and d; as well as VvSRO2b, RcSRO2b, and PtSRO2e and f. The group II (IIa and b) contains only SRO members with domain structure of type B (PARP and RST domain). Strikingly, *P. patens*, *S. moellendorffi*, rice, and *B. distachyon *do not contain proteins that cluster together with group II (Figure 6) suggesting that this group is specific for eudicots.

As described before, one to one orthology exists between the SROs from *Arabidopsis *species *A. thaliana *and *A. lyrata*, as evidenced by the tight clustering in cladograms, (Figure 1B and 1C; Figure 6). In *Arabidopsis*, *SROs *were always present in pairs consistent with a previously proposed duplication event ([2,34], Plant Genome Duplication Database http://chibba.agtec.uga.edu/duplication/index/home. Similar duplications are documented for several other gene families, e.g. the B3 DNA-binding superfamily [35]. SRO group I members of other, more distantly related plant species lacked such pairing and bore no greater similarity to either AtRCD1 or AtSRO1 but rather formed a sister branch within group I. This raises the question of when the duplications occurred. An analysis of available expressed sequence tags (ESTs) from *Brassica rapa*, *Brassica oleracea *and *Brassica napus *revealed the presence of distinguishable orthologs for AtRCD1 and AtSRO1 in *Brassica *species (Additional file 1). This suggests that the split between AtRCD1 and AtSRO1 might have occurred during the diversification of the Brassicaceae, while other plant species retained so-called "co-orthologs" to AtRCD1/AtSRO1 [36]. These refer to sister groups related equally to both proteins, which are derived from the expansion of paralogous genes in the individual species. The situation was similar for group IIa; *Brassica *contained ESTs which can be assigned as orthologous to either SRO2 or 3 (Additional file 1). In contrast, while *Brassica *group IIb orthologs were found for AtSRO5, no sequences similar to AtSRO4 were found. However, it remains unclear if this indicates the absence of an AtSRO4 ortholog from *Brassica*, or if this gene was simply missing from the current EST collections due to low expression levels.

These results demonstrate the presence of group I SROs with a conserved structure and domain architecture in all the genomes studied here and suggests their presence in all extant plant species, while group II SROs are unique to eudicot plants. Intriguingly, both monocot species analyzed possess only group I SROs. The lack of group II in members of the more basal plant groups suggests that the origin of the SROs lies within group I, and that group II represents a later development. It is possible that the group II evolved within eudicots only after the dicot-monocot split, or that at least some monocots, represented in this study by two grasses, have lost these groups after these plant lineages diverged more than 120 million years ago [37]. Resolving this question will require investigation of further genomes especially species from the basal branches of angiosperms and gymnosperms, which are not currently available. Several informative plant species, including loblolly pine (*Pinus taeda*, a coniferous gymnosperm) are currently being sequenced.

### The conservation of the RST domain between plant groups

**Figure 7:**
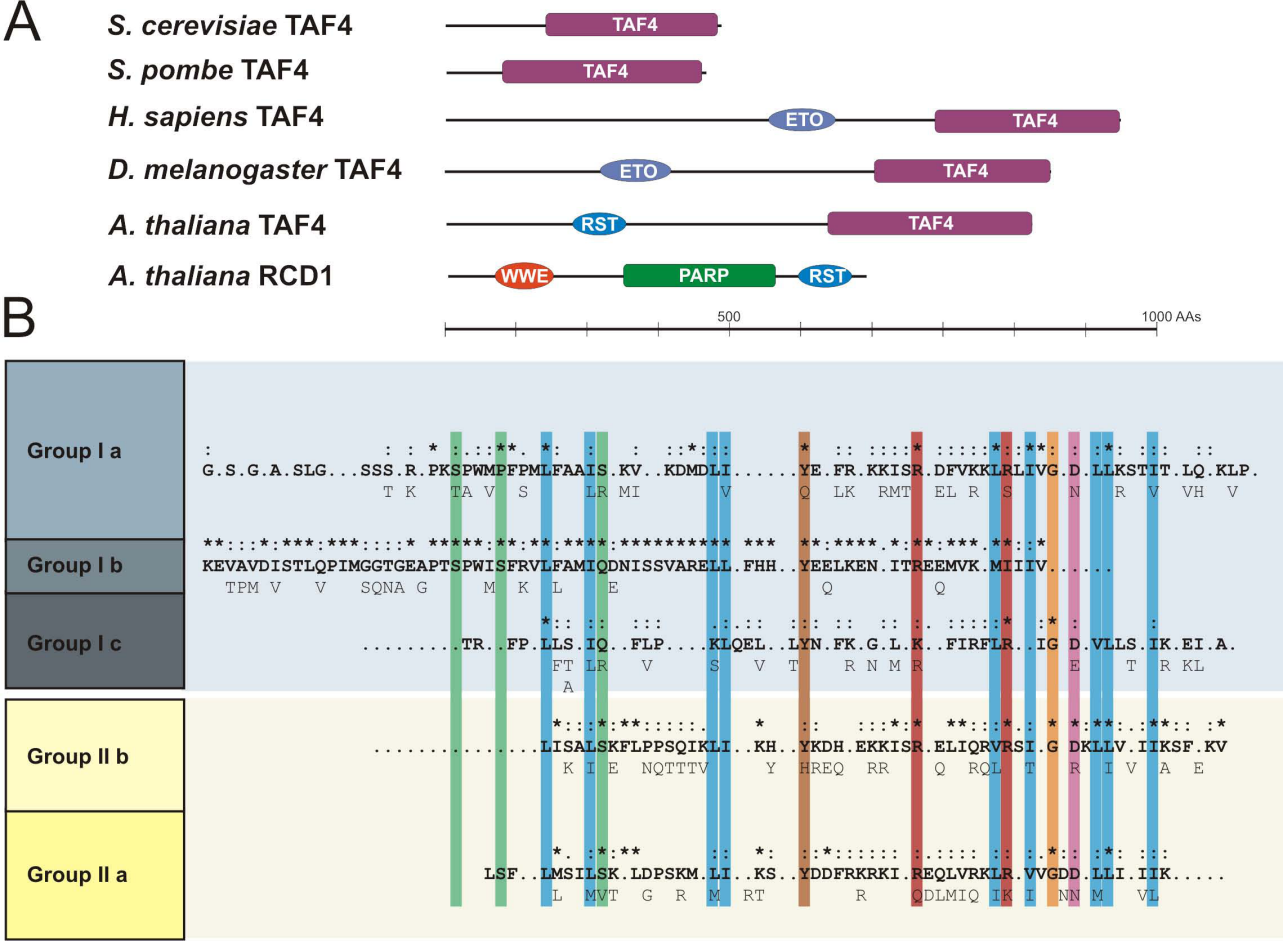
**The RST domain of the plant SRO protein family contains a strongly conserved amino acid pattern**. (A) Domain structure of AtRCD1 and TAF4s from multiple species (*Saccharomyces cerevisiae*, *Schizosaccharomyces pombe*, *Homo sapiens*, *Drosophila melanogaster*). All TAF4s have the conserved TAF4 superfamily domain (TAF4; PF05236). Yeast TAF4s lack an N-terminal extension while metazoan TAF4s have an extension bearing an ETO domain (ETO/TAFH domain; PF07531), which is a known transcription factor-recruitment domain [43]. Plant TAF4s also have an N-terminal extension that lacks the ETO domain but bears the structurally unrelated plant-specific RST (RCD1-SRO-TAF4; PF12174) domain. TAF4 RST has not been tested for TF interaction, however, the RST domain from AtRCD1 is required for interaction with multiple TFs. AtRCD1 also bears PARP-like (PS51059) and WWE (PS50918) domains. (B) The C-terminal RST domain of the different groups and subgroups (Ia, Ib, Ic, IIa, IIb) of the plant SRO protein family were aligned using ClustalW and Boxshade. Consensus sequences for each group or subgroup are depicted in bold characters and marked according to similarity: conserved (*), strong similarty (:), weak similarity (.) using Boxshade. Under the sequence, alternatives for AAs are shown. AAs with similar chemical properties are indicated using colored bars. Green indicates polar, non-charged, non-aliphatic residues. Blue indicates the most hydrophobic AAs. Red indicates positively charged AAs. Magenta highlights acidic residues. Orange shows glycine and brown indicates tyrosine.

A novel conserved domain in the C-terminus of plant SROs was identified recently [1]. This RST domain is also present in TAF4 (Figure 7A), which is a component of several multimeric protein complexes including primarily the general transcription factor TFIID involved in transcriptional initiation [38,39]. The RST domain is distinct from the conserved TAF4 superfamily-defining domain (PF05236), which is required for the assembly of the TFIID complex (Figure 7A; [1,38]). Here the analysis of the RST domain has been expanded, demonstrating that it is present in all known SRO family members (Figure 5). In the few cases of SROs without an RST domain, the gene annotation was questionable and requires further verification through mRNA support for the gene model (see Methods).

Alignments of the C-terminus of SRO family members from different plant species, representing all groups and subgroups, demonstrated that the RST domain is universally conserved (Figure 7B). The SRO group I was subdivided into three subgroups (Ia, Ib and Ic) based on the sequence of the PARP domain (Figure 6) and analysis of the RST domains resulted in the same grouping (Figure 7B). Members of the groups Ia and Ib have an approximately 20 AA long extension in the N-terminus of the RST domain compared to the members of the groups Ic and II. Since the group Ib contains SROs from *P. patens *together with SROs from grasses and the eudicots castor bean and poplar, it might represent an ancestral SRO group. A strong conservation of a large number of aliphatic AAs in the N- and C- termini of the RST domain, with a strictly conserved tyrosine in the middle of the domain and two conserved positively charged AAs in the second half of the domain, was striking (Figure 7B). The strong conservation of aliphatic AAs in the C-termini of the SRO proteins points to a conserved alpha-helical structure. This sequence preservation implies strong functional constraints for the RST domain during the diversification of the SRO protein family, possibly ensuring that a critical structure of the SRO C-terminus is retained in spite of sequence divergence.

### The functional domains of the A. thaliana SRO proteins

#### The RST domain mediates transcription factor interactions

**Figure 8:**
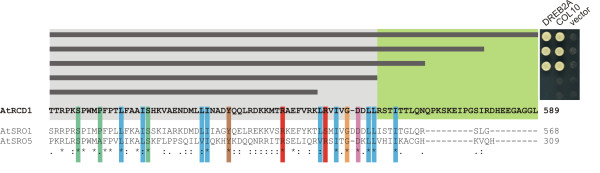
**The RST domain of AtRCD1 is required for the TF interactions**. The C-terminus of AtRCD1 was truncated to determine the minimum protein length capable of interacting with TFs. The dark gray horizontal bars above the AtRCD1 protein sequence denote the different constructs. Green background indicates interaction and gray background the lack of it. Yeast spots from each interaction test are depicted in the panel on the right. The ClustalW alignment of AtRCD1, AtSRO1 and AtSRO5 is included for comparison of the RST structure in different proteins. Highlighted AAs in the protein sequences are as in figure 7.

AtRCD1 interacts with several transcription factors (TFs) in the yeast 2-hybrid system (Y2H) and *in vitro*. The WWE and PARP domains are dispensable for these interactions [1,3]. Analysis of mutants lacking the RST domain of AtRCD1 and AtSRO1 demonstrated the significance of this TF-interacting domain for plant development and stress responses. In contrast to AtRCD1, AtSRO1 only interacts with a subset of these TFs [1]. The C-terminus of AtRCD1 is 18 AAs longer than that of AtSRO1 and thus could account for its broader range of TF interactions. To further characterize the RCD1-TF interactions, we constructed C-terminal truncations of AtRCD1 and tested them for interaction with DREB2A and COL10 (Figure 8), two known AtRCD1 interacting TFs [1]. Deletion of the 18 AA extension did not affect the RCD1-TF interactions and also the next three AAs (Q569-K517) were dispensable. However, deletion of further nine or more AAs (N568-L559), which extend into the conserved RST domain, disrupted interactions supporting the proposed role for RST as a functional protein interaction domain. AA D552 in AtSRO1 is absent from AtRCD1 (and all other SROs), and was thus another candidate for the observed differences in the interactions. However, deletion of this residue did not affect the AtSRO1-TF interactions (data not shown). Thus, the determinants of interaction specificity must lie in the other residues within the RST domain or elsewhere in the protein.

**Figure 9:**
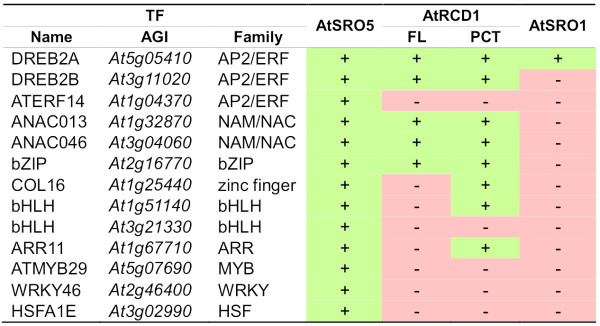
**AtSRO5 interacts with transcription factors**. Transcription factors interacting with AtSRO5 in a pairwise interaction test against the REGIA TF collection. FL: Full length AtRCD1. PCT: AtRCD1 construct lacking the WWE domain. + interaction observed, - interaction not observed.

To address if the conserved SRO5-RST domain is also a TF-interaction domain we screened the REGIA (TF) collection [1,40] with AtSRO5, a group IIb SRO, which has been shown to be involved in salt stress responses [18]. AtSRO5 interacted with 13 TFs out of the more than 1300 present in the collection (Figure 9). Three TFs belong to the AP2/ERF TF family and two to the NAM/NAC and bHLH families each. Five of these TFs interact also with AtRCD1, and DREB2A with both AtRCD1 and AtSRO1 [1]. In addition, AtSRO5 interacted with 3 proteins that were not recovered with full-length AtRCD1 but interacted with a truncated version, which lacks the WWE domain (PCT), thus resembling the AtSRO5 domain structure. Three TFs (AtMYB29, WRKY46 and HSFA1E) were unique interaction partners for AtSRO5, although AtRCD1 interacted with other members of the same TF families [1]. AtSRO5 was previously reported to localize to mitochondria [18]. However, bioinformatic prediction of its subcellular localization rather suggested a different targeting of the protein. This, together with the multiple TF interactions of AtSRO5 prompted us to investigate the cellular distribution of the AtSRO5 protein.

**Figure 10:**
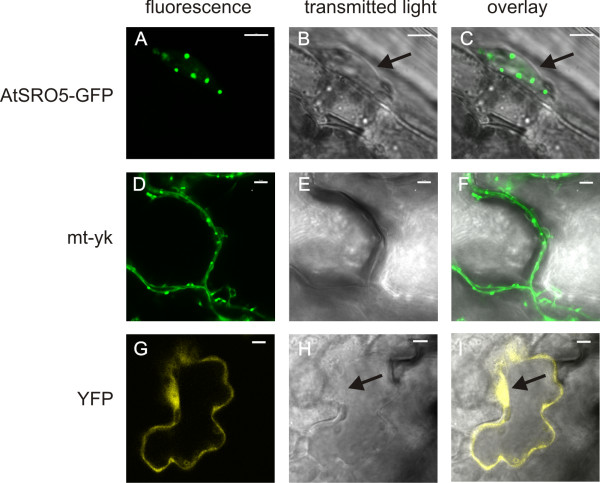
**Subcellular localization of AtSRO5**. The AtSRO5-GFP fusion protein localized to several dot-like structures in the nucleus in *A. thaliana *seedlings (panels A-C). As comparison, the mitochondrial localization marker line mt-yk (panels D-F) and the nuclear and cytoplasmic localization of YFP protein (panels G-I) are shown. Panels A, D and G display the fluorescent signal, panels B, E and H the light micrograph and panels C, F and I the two overlaid. Scale bar 5 *μ*m; arrows in B, C, H and I indicate the nucleus.

Ectopic expression of AtSRO5-GFP in *A. thaliana *seedlings showed that AtSRO5 localized to several dot-like structures in the nucleus (Figure 10, panels A-C). The results were verified by transient expression of the same construct in *Nicotiana benthamiana *leaves (data not shown). The difference in the observed subcellular localization could be due to the use of different expressions systems. Thus we cannot exclude that AtSRO5 localizes to mitochondria under certain conditions.

These results give possible biological relevance to the interactions between AtSRO5 and TFs. Constant communication between the mitochondria and the nucleus is required for normal cellular function [41]. AtSRO5 might participate in bidirectional interorganellar signaling and play a role in regulating nuclear gene expression through the TF interactions. However, the implications of AtSRO5 localization to other cellular compartments in addition to the mitochondria require further studies to reveal its significance.

The high number of TF interactions in the Y2H screen demonstrates functional conservation of the RST domain and its importance for protein-protein interaction (this study, [1]). The RST domain is also present in plant TAF4 proteins. Human and *Drosophila *TAF4s have an N-terminal extension carrying the ETO-TAFH domain (Figure 7A). This domain recruits various transcription factors to the TFIID initiation complex and thereby participates in the regulation of transcription [42,43]. The ETO-TAFH domain is missing from plant TAF4 proteins; instead, the TAF4 N-terminus bears the RST domain (Figure 7A). Its presence and position in relation to other domains suggests that the RST domain could be functionally equivalent to other, animal specific, TF-interaction domains. Strong conservation between the RST domains from TAF4 and the SROs could hint towards a common function of TF binding. TF recruitment to TFIID by TAF4 RST is a paradigm for transcriptional regulation. Competition for, or modification of, common TF interaction partners is a model for the modulation of TAF4 dependent processes by the SROs. The future challenge will be to resolve the structure of several highly similar RST domains including AtRCD1, AtSRO1, AtSRO5 and also TAF4s. This together with mutagenesis and deletion studies based on the comparisons (Figures 8 and 9) will help to understand the basis of the specificity of the TF interactions. *In planta *verification of the interactions and competition experiments between SROs, TFs, and TAF4s will be required to determine the significance of the protein-protein interactions.

#### The conserved PARP domain in SRO-proteins: structural vs. functional conservation

**Figure 11:**
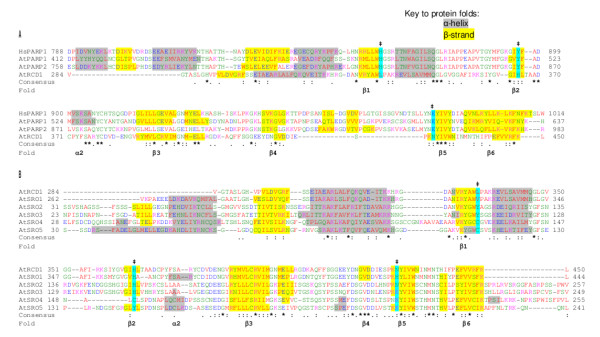
**Conserved active site fold structure of the PARP domain**. Fold-assisted AA alignments of the PARP catalytic core from (A) human PARP1 (HsPARP1), *A. thaliana *PARP-1 and -2 (AtPARP1, AtPARP2) and RCD1 (AtRCD1) and (B) *A. thaliana *RCD1 and SROs (AtSRO1-5). Consensus of conserved (*) and similar (: and .) AAs and conserved folds (*α*-helix or *β*-sheet) are indicated below the alignments. Additionally folds are shaded in the alignment with grey (*α*-helix) or yellow (*β*-sheet) backgrounds. Conserved ADP-ribosyl transferase catalytic triad, composed of three AAs at the C-terminus of *β*-sheet 1, middle of *β*-sheet 2 and N-terminal end of *β*-sheet 5, is indicated by turquoise background shading and an (‡) above the alignment. Alignments were performed with T-Coffee at EMBL-EBI http://www.ebi.ac.uk/Tools and hand-adjusted according to fold predictions performed with Psipred in the Phyre search [53]. AAs were color-coded according to their biochemical properties as in http://www.ebi.ac.uk/Tools/t-coffee/help.html#color.

Based on the presence of a PARP catalytic domain, it has been presumed that *A. thaliana *RCD1 and SRO proteins could have ADP-ribosyl transferase activity [1,2,6], which seems to be confirmed by the conserved fold structure (Figure 11). The alignment of AtPARPs and AtRCD1 with HsPARP1, for which the 3D structure has been solved, allowed for identification of conserved fold structures as landmarks in *A. thaliana *PARPs (Figure 11A). Generally, the fold structure is well conserved and all of the folds that constitute the active site are present (*β *sheets 1-6 and *a *helix 2, Figure 11A). Some additional plant specific folds not present in the HsPARP1 are predicted in AtPARP1 and 2, AtRCD1 and AtSROs (Figure 11A and 11B). These additional predicted features, if present, apparently do not disrupt the activity in AtPARP1, which was shown to exhibit PARP activity (Table 2; [44]). The conserved active site folds also mark the position of the catalytic triad, the three AAs histidine (H), tyrosine (Y) and glutamic acid (E), which is conserved in AtPARP1 and 2 but not AtRCD1 or AtSROs (Figure 11A and 11B; Table 2). The H333 to L and Y365 to H substitutions at the NAD binding sites within the HYE catalytic triad of RCD1 (Table 2) suggest that it has lost the ability to bind NAD.

**Table 2:** Characteristics of the putative SRO active sites.

Name	Identifier	WWE	Catalytic Core Motif	Loop length (between *β*4 and *β*5)	NAD binding	Predicted Activity	Confirmed Activity
AtPARP1	At4g02390.1	No	H486 Y520 E614	38	yes	PARP	PARP [44]

AtPARP2	At2g31320.1	No	H833 Y867 E960	36	yes	PARP	ND

AtPARP3	At5g22470.1	No	C653 V687 E782	36	yes	PARP	ND

AtRCD1	At1g32230.1	Yes	L333 H365 N428	5	no *	inactive	inactive *

AtSRO1	At2g35510.1	Yes	V329 H361 N422	5	ND	inactive	ND

AtSRO2	At1g23550.1	Yes	Y118 H153 N216	5	ND	inactive	ND

AtSRO3	At1g70440.1	Yes	Y110 H145 K208	5	ND	inactive	ND

AtSRO4	At3g47720.1	Yes	C129 C150 K214	6	ND	inctive	ND

AtSRO5	At5g62520.1	Yes	C113 Y143 K207	5	ND	inactive	ND

HsPARP1	P09874	No	H862 Y896 E988 [24]	37	yes	PARP	PARP

HsPARP7	Q7Z3E1	Yes	H532 Y564 I631 [26]	6	ND	mART	ND

HsPARP10	Q53GL7	No	N886 Y919 I987 [26]	6	yes	mART	mART

HsPARP11	Q9NR21	Yes	H197 Y229 I313 [26]	6	ND	mART	ND

HsPARP12	Q9H0J9	Yes	H564 Y596 I660 [26]	6	ND	mART	ND

HsPARP13	Q7Z2W4	Yes	Y787 Y819 V876 [26]	6	ND	inactive	inactive

HsPARP14	NP_060024	Yes	H1682 Y1714 L1782 [26]	6	yes	mART	mART

Relationship between conserved AA motif of the ADP-ribosyl transferase catalytic triad, the loop length between *β*-sheets 4 and 5, and predicted or know catalytic activity of selected *A. thaliana *and human PARPs and SRO family members. Presence or absence of the WWE domain (PS50918) and catalytic activities are indicated. Results of this study are marked with an asterisk (*).

To test the predictions of activity based on the fold structure of the PARP domain, we expressed the *A. thaliana *full length RCD1 protein and a truncated form containing the PARP and RST domains (PCT; AAs 241-589) as GST-tagged proteins in *Escherichia coli*. The recombinant proteins were partially purified by affinity chromatography with glutathione sepharose and used for testing NAD binding. *Pisum sativum *short-chain alcohol dehydrogenase-like protein A (SAD-A, [45]) was used as positive control.

NAD binding was investigated by covalent cross-linking of bound NAD by ultraviolet irradiation [46,47]. After UV irradiation of sample mixtures containing radioactive NAD and the proteins tested, the proteins were separated by SDS-PAGE and labeling with [*α*-^32^P-NAD] was monitored by autoradiography. To verify the specificity of NAD binding, competition experiments were performed with excess of unlabeled NAD.

**Figure 12:**
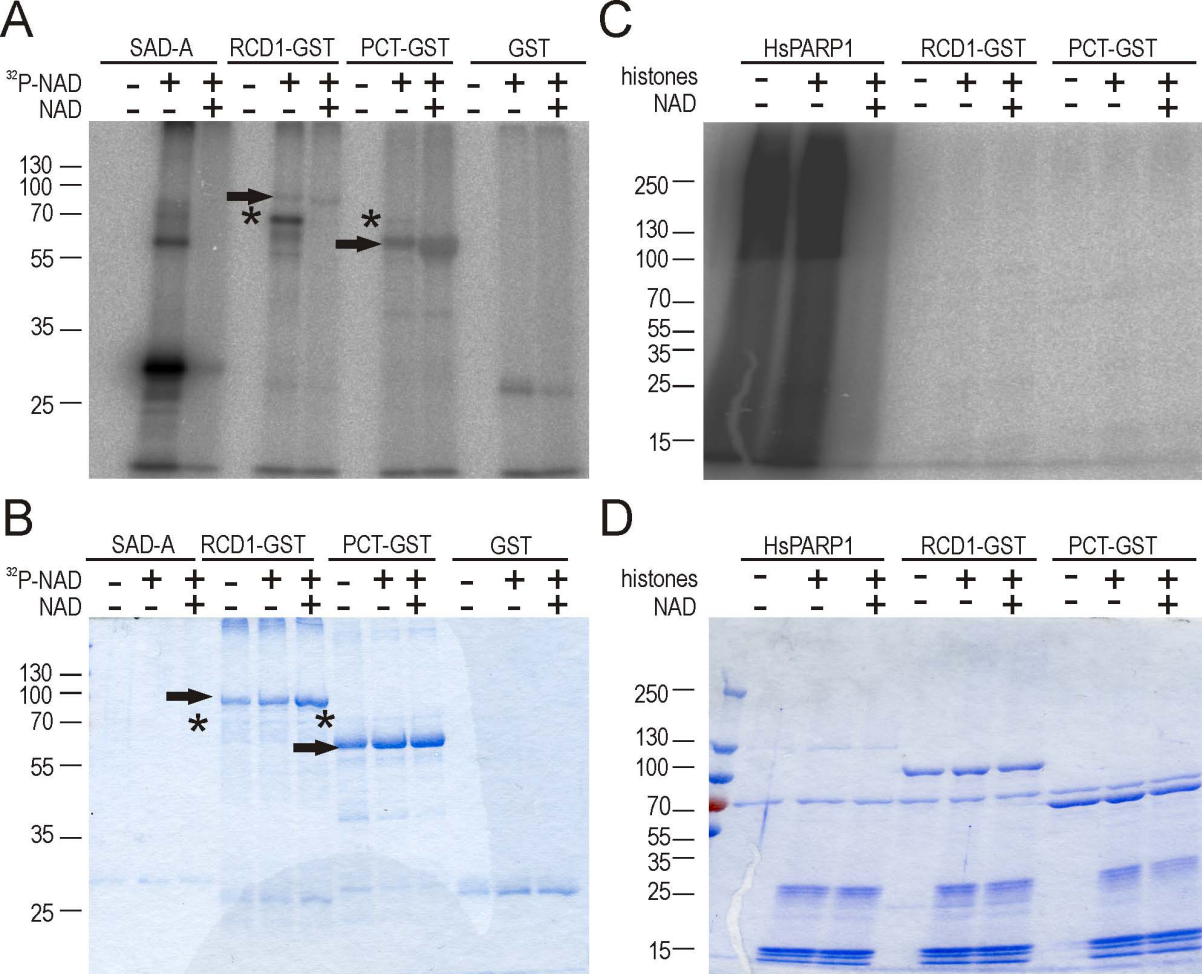
**AtRCD1 does not bind NAD and does not have ADP-ribosylation activity**. Biochemical analysis of NAD binding and ART activity of AtRCD1. (A) NAD binding analysis: Autoradiography image of a SDS-PAGE gel showing proteins labeled with [^32^P-NAD] upon UV irradiation. SAD-A, RCD1-GST, PCT-GST or GST were incubated with 0.6 *μ*M of [^32^P-NAD] in absence or presence of 0.6 mM of unlabeled NAD under UV light (see Methods). (B) Picture of the SDS-PAGE gel shown in (A) stained with Coomassie Brilliant Blue. Positions of RCD1-GST and PCT-GST are marked on panels (A) and (B) with arrows; asterisks mark the position of the DnaK protein. (C) ART activity analysis: Autoradiography image of SDS-PAGE gel showing poly-ADP-ribosylation of proteins in presence of [^32^P-NAD]. HsPARP1, RCD1-GST or PCT-GST in concentration 200 nM were incubated with 1.3 *μ*M [^32^P-NAD] (see Methods) in absence or presence of 3 *μ*g of histones. (D) Picture of the SDS-PAGE gel shown in (C) stained with Coomassie Brilliant Blue. The 70 kDa band represents BSA used as a carrier for protein precipitation. All panels: Unlabeled NAD was used in the competition experiments. Molecular weight marker sizes (kDa) are indicated on the left side of each panel. The experiment was repeated three times with similar results, one representative experiment is shown.

The NAD binding of the positive control, SAD-A, was visible as two bands in an autoradiogram (Figure 12A). The major band at 30 kDa corresponds to monomeric form of the enzyme, and the minor band at 60 kDa to the dimer [45]. The presence of 1000-fold excess of unlabeled NAD resulted in the disappearance of both bands, indicating that the NAD binding was specific (Figure 12A). In contrast, RCD1-GST, PCT-GST and GST did not bind NAD (Figure 12A). The weak bands visible on the autoradiogram at the molecular weights corresponding to RCD1-GST or PCT-GST (indicated by arrows in figures 12A and 12B) or GST alone, respectively, did not disappear in presence of unlabeled NAD, indicating unspecific labeling of the proteins. The 70 kDa band visible in the autoradiogram (Figures 12A and 12B, asterisk) represented a contaminant in the purified RCD1-GST and PCT-GST samples. It was identified by mass spectrometry as DnaK molecular chaperone from *E. coli*. DnaK protein contains a nucleotide-binding domain explaining its ability to bind NAD.

These results demonstrated that AtRCD1 does not bind NAD and thus should not have ART activity. To verify this, we tested possible poly(ADP-ribosyl) transferase activity of RCD1-GST and PCT-GST directly in a standard ART activity assay using recombinant HsPARP1 as a positive control. HsPARP1 exhibited automodification (Figure 12C, a smear at molecular mass above 116 kDa) but no auto-poly(ADP-ribosyl) transferase activity was detected for RCD1-GST or PCT-GST (Figure 12C and 12D). Possible substrate modification by RCD1-GST or PCT-GST was analyzed by supplementing the reaction mixture with histones, which are classical PARP targets. Neither RCD1-GST nor PCT-GST exhibited detectable PARP or mART activity (Figure 12C and 12D). Additionally, DREB2A, the most prominent RCD1 interaction partner, could be a possible substrate [1]. However, no PARP or mART activity of RCD1-GST or PCT-GST towards DREB2A-GST was detected (data not shown).

In light of these results, it is remarkable that the SROs structurally resemble PARPs/ARTs so closely. It may be possible that the PARP domain of AtRCD1 and the SROs still has an activity related to ADP-ribosylation. A novel mechanism has been described for HsPARP10, which lacks the catalytic glutamic acid (E), the third conserved AA of the catalytic triad (Table 2). HsPARP10 has still retained mART activity via a novel mechanism in which the active E is provided by the substrate protein [24]. HsPARP10 has a shorter linker sequence between folds *β*4 and *β*5 [24] which facilitates an open active site configuration necessary for the substrate glutamic acid entry into the active site. AtPARPs (AtPARP1, 2, and 3) retain a long *β*4-*β*5 linker but all AtSROs have the shorter linker (Table 2) suggesting a more open active site fold. The bioinformatic analysis revealed the loss of both conserved NAD contacting H and Y in the *A. thaliana *SRO PARP domains making such a substrate-mediated mART activity unlikely (AtSRO5 is an exception to this, it has lost the H but retained the Y). This is supported by our biochemical analysis which demonstrated that AtRCD1 is not able to bind NAD, and, consequently, does not have mART or PARP activity. Other similar changes in the catalytic triad of the other AtSROs suggest they too may lack the capacity for NAD binding and ART activity (Table 2). Interestingly, this is also true for active sites in SROs from other plant species (Additional file 2), with the notable exception of *P. patens *SROs, which bear more conserved and potentially active catalytic triads.

## Conclusions

The SROs are a protein family with a unique domain architecture which is conserved in all land plants. The SRO proteins can be subdivided into two groups representing two different structural types. Different plant groups have experienced expansion of different SRO groups during evolution. Interestingly, the basal plant groups, *P. patens*, a moss, and *S. moellendorffi*, a lycopodiopsid, as well as monocots possess only group I SROs, while eudicots additionally contain group II SROs. Our analysis suggests that the evolutionary origin of the SROs lies within subgroup Ib, which could be ancestral to all other SROs. Alternatively, monocots and more basal vascular plants might have experienced a secondary loss of group II SROs.

While the N-terminal WWE domain is only present in group I SROs of the structural type A, virtually all SROs analyzed contain a PARP-like domain and a C-terminal RST domain (Figure 7B). The conservation of the C-terminus of the SROs suggests functional constraints and a subsequent requirement for the conservation of a particular structure (Figure 7B). A possible function is the interaction with transcription factors (Figure 8), which has been demonstrated for several *A. thaliana *SROs, including AtSRO5. For a protein localized to mitochondria [18], its ability to interact with several transcription factors in Y2H analysis was unexpected. Our analysis of subcellular localization for AtSRO5 showed that the protein is localized to several dot-like structures in the nucleus (Figure 10) which supports the significance of the TF interactions. Nevertheless, it is possible that AtSRO5 localizes to the mitochondria under certain conditions linking TF interactions to retrograde signaling and mitochondrial metabolism [48].

The PARP-like domain is the most conserved feature of the SROs. However, based on bioinformatic and biochemical evidence (Figures 11 and 12), we suggest that the SROs do not possess PARP or mART activity. Nevertheless, the fold structure of the PARP-like domain is highly conserved (Figure 11). As a comparison, it is estimated, that 10% of the receptor-like protein kinases encoded in the *A. thaliana *genome are inactive but nevertheless expressed and translated and potentially function as co-receptors [49]. What other possible function or activity might those PARP/ART-like domains possess? The structural conservation of an enzymatically inactive domain could facilitate complex formation or stabilization and be an advantage for the organism. Regardless of which activity is eventually discovered in the SROs, they have important functions in plant stress responses and in development.

## Methods

### Sequence identification and phylogenetic analysis

Protein sequences for SROs of species used in this study were obtained from the respective projects databases (see Table 1 for reference) using HMMER and BLAST searches. Additionally, the genomes of aquatic, photosynthetic, and plant associated microorganisms were queried, including the green algae *Chlamydomonas reinhardtii *and *Ostreococcus tauri*; the yeasts, *Saccharomyces cerevisiae *and *Schizosaccharomyces pombe*; the plant pathogenic fungi, *Magnaporthe grisea *and *Botrytis cinerea*; as well as the photosynthetic cyanobacteria *Rhodobacter sphaeroides *and *Synechocystis *sp. The genomes of these microorganisms did not contain genes related to SROs.

The assembly scaffold of the *A. lyrata *genome was a kind donation of Prof. Detlef Weigel. *A. lyrata RCD1-SRO *orthologs were identified by genomic blast with the *A. thaliana RCD1-SRO *genomic sequences. The *A. lyrata *sequences were subsequently spliced according to the *A. thaliana *gene models and converted to protein sequences. Some genomes were excluded due to gene models of SRO protein family members with significant dissimilarity to *A. thaliana *gene models and lack of cDNA support for these unique gene models.

The protein domains were identified using SMART [32,33] and ScanProsite [30,31]. cDNA sequences and ESTs were obtained *via *BLAST search through the NCBI webpage http://www.ncbi.nlm.nih.gov/. Sequences were, if possible, verified for being full length by comparison to existing ESTs from available collections. Some gene models were included for completeness; however, their dissimilarity to *A. thaliana *SROs and lack of cDNA support made them questionable: the gene models for OsSRO1d and OsSRO1e predicted long C-terminal extensions but ESTs suggested that OsSRO1d ended in the PARP domain and OsSRO1e contained a RST domain of normal length. PpSRO1a and PpSRO1b sequences were likely to be incomplete as the PARP domain extended until the end of the predicted protein. PpSRO1c contained a long C-terminus but ESTs suggested a shorter protein similar to other SROs. The C-terminal part of SmSRO1a from *S. moellendorffi *showed only moderate similarity to the C-terminus of other SROs. The annotation predicted a long C-terminal extension but EST support suggested only a short C-terminal domain. Due to the lack of other SRO sequences from organisms more closely related to *S. moellendorffi*, we were unable to determine if the C-terminus of SmSRO1a represented a unique development or a misannotation. Two additional putative SROs from *S. moellendorffi *were truncated and thus could not be assigned to any group. These sequences from rice, *P. patens*, and *S. moellendorffi *will require future verification.

Sequence alignments were performed using ClustalW2 [50] and colored using the Boxshade programme http://www.ch.embnet.org/software/BOX_form.html. Subsequent phylogenetic analysis was performed using Phylip and MEGA4 [51,52].

Active site alignments were preformed with T-Coffee at EMBL-EBI http://www.ebi.ac.uk/Tools using only sequences of PARP domains as defined above. Fold predictions utilized Psipred in the Phyre search http://www.sbg.bio.ic.ac.uk/phyre[53]. Alignments were then hand adjusted with the guidance of conserved fold structures. Catalytic triad positions were determined as the positions within conserved folds corresponding to the HYE triad from HsPARP1 and AtPARP1.

### Yeast two-hybrid work

Yeast work was conducted as described in [1] using the GAL4-based ProQuest Y2H system (Invitrogen, Carlsbad, CA, USA). 10 mM 3-aminotriazole was used for eliminating autoactivation in all experiments. The primers used for cloning are described in additional file 3.

### Gene expression analysis

The *sro5-2 *allele was obtained from the GABI-Kat collection at the German Resource Center for Genome Research (line 325B05) [54]. Microarray hybridizations (4 biological repeats) and data analysis were performed as previously described [1]. qPCR experiments for gene expression analysis were done according to Wrzaczek *et al*. [55]. The primers used for qPCR are described in additional file 3.

Affymetrix raw data was downloaded from NASCArrays http://affymetrix.arabidopsis.info/narrays/experimentbrowse.pl (accession number NASCARRAYS-143, paraquat; NASCARRAYS-353, ZAT12; NASCARRAYS-176, ABA time course experiment 1; NASCARRAYS-192, Ibuprofen), ArrayExpress http://www.ebi.ac.uk/microarray-as/ae/ (accession numbers E-GEOD-12856, *Blumeria graminis *sp. *hordei*; E-GEOD-5684, *Botrytis cinerea*; E-GEOD-5743, 2,4-Dichlorophenoxyacetic acid (2,4-D); E-ATMX-13, Methyl Jasmonate; E-MEXP-550 polychromatic radiation with decreasing short-wave cut-off in the UV range (UV-B experiment); E-MEXP-739, Syringolin A; E-MEXP-1797, Rotenone), Gene Expression Omnibus http://www.ncbi.nlm.nih.gov/geo/ (accession numbers GSE5615, Elicitors LPS, HrpZ, Flg22 and NPP1; GSE5685, Virulent and avirulent *Pseudomonas syringae*; GSE9955, BTH experiment 1; GDS417 *E. cichoracearum*; GSE5530, H_2_O_2_; GSE5621, Cold time course experiment; GSE5622, Osmotic stress time course experiment; GSE5623, Salt time course experiment; GSE5624, Drought time course experiment; GSE5722, O_3_; GSE12887, Norflurazon; GSE10732, OPDA and Phytoprostane; GSE7112, ABA experiment 2) and The Integrated Microarray Database System http://ausubellab.mgh.harvard.edu/imds (Experiment name: BTH time course, BTH experiment 2).

The raw Affymetrix data was preprocessed with RMA using probe set annotations (custom.cdf files) from http://brainarray.mbni.med.umich.edu/, version 11.0.1. Biological repeats of each experiment were combined by computing a mean of the measured gene expression. Gene expression was summarized by computing a log_2 _ratio of the treatment and control expressions (differential expression, DE). A visualization of the DE values is shown in figure 2. Variation of differential expression in an experiment *e*, , was estimated by summing the variances of (logarithm of) treatment and control gene expressions.

Parametric bootstrapping was implemented by generating 1000 samples for each experiment and each gene from a Gaussian distribution with the estimated DE as the mean and  as the variance.

Bootstrap samples were discretized to down-regulated (log_2 _DE<-1), no regulation (-1 ≥ log_2 _DE ≤ 1), and up-regulated (log_2 _DE>1) genes. Bayesian agglomerative hierarchical clustering algorithm was then applied to the discretized bootstrap data [56]. The Bayesian hierarchical clustering algorithm computes the best number of clusters by Bayesian hypothesis testing. For each pair of genes (and experiments, depending on the clustering direction), the number of times they were assigned to the same cluster was computed. These gene (or experiment) similarities were then used as distances for computing the hierarchical clustering (Ward method) shown in figure 2.

### Protein localization

The localization of AtSRO5 was predicted using Predotar v. 1.03 http://urgi.versailles.inra.fr/predotar/predotar.html, TargetP 1.1 [57], WoLF PSORT [58] and MitoProtII - v1.101 [59]. None of the programs predicted mitochondrial localization. For *in planta *study of the localization, *AtSRO5 *was cloned into the pB7FWG2.0 [60] binary vector containing eGFP as C-terminal fusion to the protein using the primers described in additional file 3. YFP in pGREENII binary vector was used for nuclear and cytoplasmic localization control [1]. Three-day old *A. thaliana *seedlings were used for transient expression as described in [61]. The fluorescent proteins were visualized using confocal laser scanning microscopy after 36 hours of co-cultivation. The mitochondrial localization control line mt-yk (N16264) was obtained from Nottingham Arabidopsis Stock Centre and imaged at the same age as the transiently transformed plants.

### Protein expression and purification

Full-length AtRCD1 and its truncated version, PCT, consisting of PARP and RST domains (AAs 241-589) were cloned into pGEX4T-1 for N-terminal GST fusion using the primers listed in additional file 3. After sequencing, the constructs were transformed into the *E. coli *strain BL21 (DE3) CodonPlus RIL for protein production.

LB medium containing ampicillin (100 *μ*g ml^-1^) and chloramphenicol (50 *μ*g ml^-1^) was inoculated with 1/50 volumes of overnight bacterial culture and grown at 37°C until OD_600 _reached 0.6-0.8. Expression of PCT-GST and DREB2A-GST was induced by adding isopropyl-*β*-D-galactoside (IPTG) to a final concentration of 0.5 mM, and the culture was transferred to 28°C. After 4 hours, the cells were harvested by centrifugation at 5000 *g *and stored at -20°C.

For RCD1-GST expression, benzyl alcohol was added to the cell culture with OD_600 _0.5-0.6 to a final concentration of 10 mM and the cells were grown for additional 30 min at 22°C [62]. Protein expression was induced by 0.1 mM of IPTG. After 16 hours at 22°C, the cells were harvested by centrifugation at 5000 *g *at room temperature, resuspended in original volume of fresh LB medium without IPTG and grown for additional 2-3 hours at 22°C. Finally, the cells were harvested by centrifugation at 5000 *g *and stored at -20°C. The cell pellets were resuspended in a lysis buffer (1/20 of initial culture volume) consisting of 50 mM Tris-HCl, pH7.5, 150 mM NaCl, 5 mM DTT, protease inhibitors cocktail (Complete, Roche Diagnostics GmbH, Mannheim, Germany). The cells were lysed by addition of lysozyme (Roche) to a concentration of 0.2 mg ml^-1 ^and incubation for 30 min at 4°C with gentle shaking. Released DNA was then digested by DNase I (Roche) at final concentration of 0.02 mg ml^-1 ^in presence of 5 mM MgCl_2 _and incubation for another 30 min at 4°C. The cell lysates was clarified by centrifugation at 20000 *g *for 15 min at 4°C. The GST tagged proteins were purified by affinity chromatography using 1-ml GSTrap columns (GE Healthcare, Chalfont St Giles, UK) according to manufacturer's instructions. SAD-A-His protein was expressed and purified as described [45]. Protein concentration was determined by Bradford method using Protein Assay reagent (Bio-Rad Laboratories Inc., Hercules, CA, USA).

### UV photoaffinity labeling

Samples of total volume 30 *μ*l containing 30 pmol of protein in 50 mM Tris-HCl, pH7.5, 100 mM NaCl, 5 mM MgCl_2_, 1 mM DTT and 0.6 *μ*M of [*α*-^32^P NAD] (0.8 mCi mmol-1) (NEN, PerkinElmer, Inc. Boston, MA, USA) were incubated in a 96-well plate on an ice bath. Unlabeled NAD in concentration 0.6 mM was added to the mixtures in competition experiment. The UV irradiation was performed for 15 min as described in [46]. The proteins were then precipitated by addition of equal volume of ice-cold 22% trichloroacetic acid and incubation on ice for at least 30 min. After centrifugation for 10 min at 16000 *g *the protein pellet was washed once with cold acetone, air-dried, and resuspended in 10 *μ*l of SDS-PAGE sample buffer [63].

### In vitro PARP activity assay

Samples corresponding to 200 nM of proteins were incubated for 20 min at 22°C in assay buffer (50 *μ*l) consisting of 50 mM Tris-HCl, pH 7.5, 100 mM NaCl, 5 mM MgCl_2_, 1 mM DTT, 10 *μ*g ml^-1 ^activated DNA (calf thymus nicked DNA, Sigma Aldrich, St. Louis, MO, USA) and 1.3 *μ*M [*α*-^32^P NAD] (0.8 mCi mmol^-1^). Recombinant HsPARP1 (Sigma) was used as a positive control. 3 *μ*g of total histones (calf thymus histones, Roche) or DREB2A-GST were added as acceptor proteins. 1 mM unlabeled NAD was added in competition experiment. The reaction was stopped by addition of ice-cold trichloroacetic acid as described above. 5 *μ*g BSA were added to the reaction mixture just before protein precipitation as a carrier.

### SDS-PAGE and autoradiography

The proteins were separated on SDS-PAGE (12% or 4-15%) according to the protocol of [63]. After protein visualization with Coomassie Brilliant Blue, the gels were dried and subjected to autoradiography. The autoradiography images were analysed with Fuji BAS-1500 phosphoimager.

### In-gel digestion and mass spectrometry

In-gel digestion and sample preparation for mass spectrometry was performed as described [64]. MALDI TOF (matrix-assisted laser desorption-ionisation time-of-flight) analysis was performed on reflector mode on a Voyager DE-PRO mass spectrometer (Applied Biosystems, Foster City, CA, USA).

## Authors' contributions

PJ, KO, MW, JPV and JK designed research. PJ, KO, MW, JPV, TB, RAR and JS carried out research. PJ, KO, MW, JPV, TB, JS and JK analyzed the data. PJ, KO, MW and JK wrote the paper. All authors have read and approved the final manuscript.

## Supplementary Material

Additional file 1 **Neighbour-joining phylogenetic tree of Arabidopsis thaliana and Brassica SROs**. The gene duplication leading to the *AtRCD1/AtSRO1*, *AtSRO2/AtSRO3 *and *AtSRO4/AtSRO5 *gene pairs in *Arabidopsis *is also present in *Brassica*. Individual protein-coding ESTs from *Brassica *can be assigned to *AtRCD1 *or *AtSRO1*, *AtSRO2 *or *AtSRO3 *or *AtSRO5*. No EST was identified for *AtSRO4*. This indicates that the gene duplication event leading to the gene pairs occurred early during the evolution of the Brassicaceae family before the split between the *Arabidopsis *and *Brassica *genera. Representative ESTs from *Brassica napus*, *Brassica rapa *and *Brassica oleracea *coding for SRO proteins were extracted via NCBI blast and the PARP domain was identified using Prosite. The PARP domains of the *Brassica *SROs were aligned with the PARP domains of the members of the *A. thaliana *and *A. lyrata *SRO protein families an unrooted Neighbour-joining tree was constructed using MEGA4. Click here for file

Additional file 2 **Active site alignments for all plant SROs**. Alignments of the region around the active site catalytic triad of all SROs analyzed here. Alignments were hand-adjusted according to the positions of conserved folds in AtRCD1, AtPARP1 and AtPARP2 as in figure 10, only the regions immediately surrounding the predicted catalytic amino acids (highlighted in red) are presented. Click here for file

Additional file 3 **Primers used for analysis**. All primer sequences used for qPCR anaylsis in the manuscript are listed in this file. Click here for file
