# Supplementary figures and images for: The RST and PARP-like domain containing SRO protein family: analysis of protein structure, function and conservation in land plants
Source: BMC Genomics. 2010 Mar 12;11:170. doi: 10.1186/1471-2164-11-170 (PMC2848248; doi:10.1186/1471-2164-11-170)

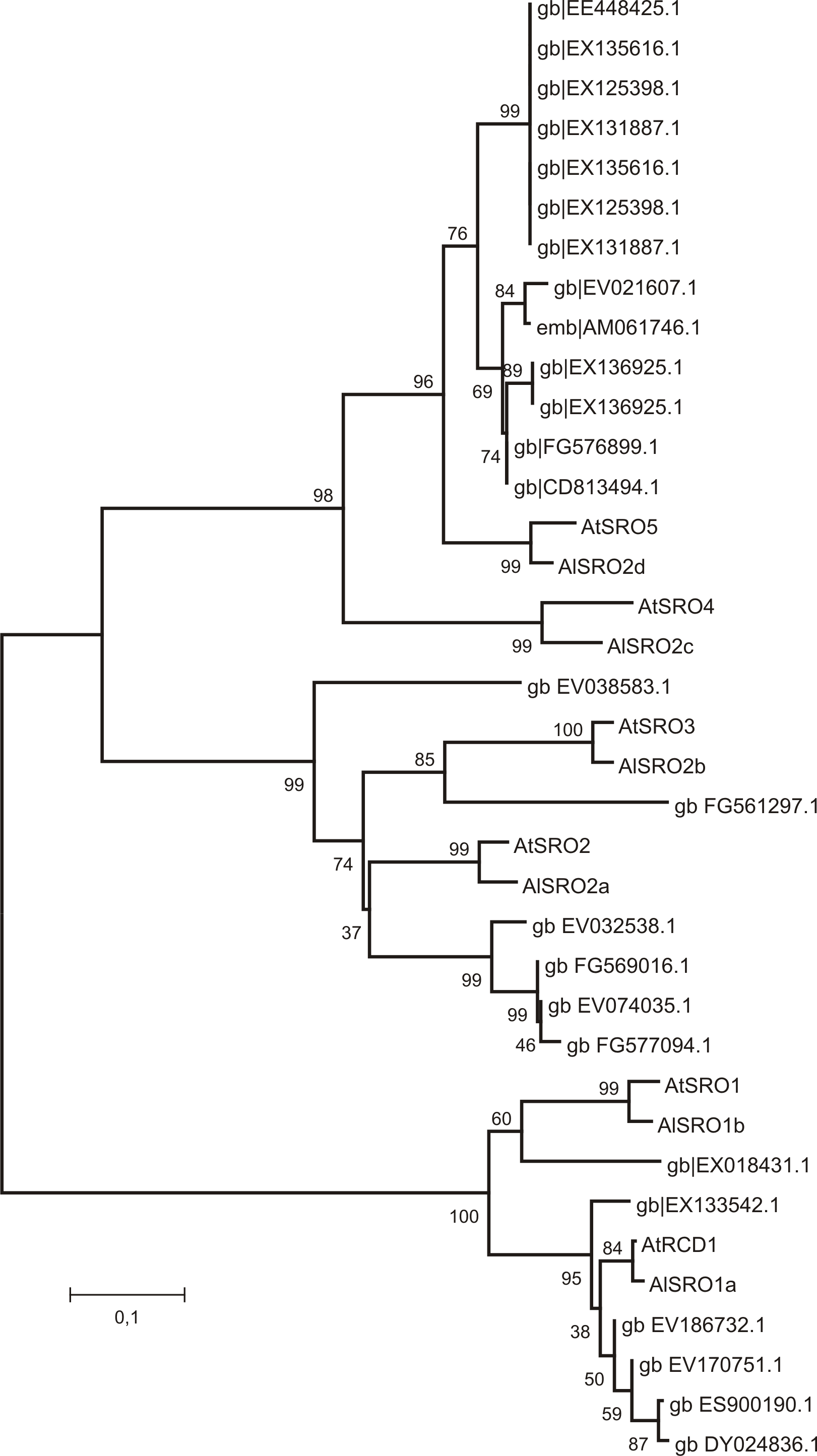

Supplement: Additional file 1 — Neighbour-joining phylogenetic tree of Arabidopsis thaliana and Brassica SROs. The gene duplication leading to the AtRCD1/AtSRO1, AtSRO2/AtSRO3 and AtSRO4/AtSRO5 gene pairs in Arabidopsis is also present in Brassica. Individual protein-coding ESTs from Brassica can be assigned to AtRCD1 or AtSRO1, AtSRO2 or AtSRO3 or AtSRO5. No EST was identified for AtSRO4. This indicates that the gene duplication event leading to the gene pairs occurred early during the evolution of the Brassicaceae family before the split between the Arabidopsis and Brassica genera. Representative ESTs from Brassica napus, Brassica rapa and Brassica oleracea coding for SRO proteins were extracted via NCBI blast and the PARP domain was identified using Prosite. The PARP domains of the Brassica SROs were aligned with the PARP domains of the members of the A. thaliana and A. lyrata SRO protein families an unrooted Neighbour-joining tree was constructed using MEGA4. [file 1471-2164-11-170-S1.PNG]
