# Supplementary material for: The RST and PARP-like domain containing SRO protein family: analysis of protein structure, function and conservation in land plants
Source: BMC Genomics. 2010 Mar 12;11:170. doi: 10.1186/1471-2164-11-170 (PMC2848248; doi:10.1186/1471-2164-11-170)
Supplement: Additional file 2 — Active site alignments for all plant SROs. Alignments of the region around the active site catalytic triad of all SROs analyzed here. Alignments were hand-adjusted according to the positions of conserved folds in AtRCD1, AtPARP1 and AtPARP2 as in figure 10, only the regions immediately surrounding the predicted catalytic amino acids (highlighted in red) are presented. [file 1471-2164-11-170-S2.PDF]

Group	Sequence			
Group I a	AtRCD1	VRYAWLPA-KREV	YGVGIHLTAAD	IESPKNYIVWN
	AlSRO1a	VRYAWLPA-KREV	YGVGIHLTAAD	IENPKNYIVWN
	AtSRO1	IRYAWVPA-KKEV	YGVGVHAANCP	VESPKHYLIWN
	AlSRO1b	IRYAWVPA-KKEV	YGVGVHAANCS	VESPKHYLIWN
	VvSRO1a	VRYAWLAS-SKEA	YGIGVHLTAVN	LQNPRHYIVWN
	VvSRO1b	VKYAWLAA-SKEA	YGIGVHLTAAN	LQNPKHYIIWN
	PtSRO1a	VRYAWLAS-SKGA	HGIGVHLSAAN	LESPREYIVWN
	PtSRO1b	VRYAWLAS-SKGA	HGIGVHLSAAN	LTSPKKYILWS
	OsSRO1a	VRYAWLAC-SKDA	YGIGTILAPAN	IKNPQHYIVWD
	BdSRO1a	IRYAWLPC-SKDT	YGAGTLLAPAN	IKNPQHYIVWD
	OsSRO1b	ERYAWLAC-TKGT	YGVGAHLAPAN	LENPRWYVVWS
	BdSRO1b	VRYAWLPC-SRDA	YGIGTHLAPAN	LQKAKHYIIWD
	RcSRO1a	CVILYSPA-KRHS	IDVPLELCSLS	LQNPGQYVVWN
Group I b	OsSRO1d	VRYGWLGS-RKQD	MRTGVYLSPEN	CSNPSYYVIWP
	OsSRO1e	VRYGWLGS-RKND	LSAGVYLSPED	LQRPKHYIIWD
	BdSRO1d	VRYGWLGS-RKSD	LSAGVYLSPED	CSNPKCYVVWP
	BdSRO1e	VRYGWLGS-TKTD	LSAGVYLSPED	CSNPKCYVMWP
Group I c	PpSRO1a	VRYGWHGT-SKKG	YGVGVYLAVEN	TISPKRLIVWS
	PpSRO1b	LRFGWHGT-SKKA	YGVGVYLACSF	INSPKRLIVWS
	PpSRO1c	VRFAWHGT-SKAG	YGVGVYLAPEE	LVSPKRLIVWS
	SmSRO1a	LQQAWYGC-SRNE	YGVGIYLSPES	LANPKRFIVWS
	RcSRO1b	AIYAWYGA-SVKD	YGVDVYLSPVG	TVNPKWYVVWP
	PtSRO1c	TVYAWYGA-PAKE	YGVGVYLSPFG	PKNPKWYVVWS
	OsSRO1c	AKFAWYGA-PAAD	HGDGVHLSPPQ	CSNPKCYVMWP
	BdSRO1c	AKFAWYGA-PSVD	HGDGVHLSPPQ	IQNPKWYVVWS
Group II a	PtSRO2d	VKYGWYSG-SKEG	HGVGVYLSPTS	LEAPRRLVVWT
	PtSRO2c	IMYGWYGG-SKEE	HGVGVYLSPTN	LEAPRRLVVWS
	PtSRO2b	VDHGWFGA-SKEE	HGLGVYLSPFE	LEAPRRLIVWS
	PtSRO2a	VKCGWYGA-SKQE	HGVGVYLSTSK	LEDPSRLVVWS
	RcSRO2a	VQFAWYGS-SREE	HGIGIHLSPAG	LHKPRRYIIWN
	VvSRO2a	INYAWYGA-SRGE	YGFGVYLSSAK	VSAPRRYIIWS
	AlSRO2b	VKYGWYSG-SKEE	HGVGIHLVHHR	LENPRKYVIWS
	AtSRO3	IRYGWYSG-SKEE	HGVGIHLVHHR	LENPRKYVIWS
	AlSRO2a	VKYGWYAG-SKPE	HGIGIHLVPSK	LHNPRNYVIWS
	AtSRO2	VKYGWYAG-SRDE	HGIGIHLVPSK	LHNPRNYVIWS
Group II b	PtSRO2f	VKFGWYGG-TRDE	YGSGIYLSPDD	LSSPKKYIVWS
	PtSRO2e	VKFGWYGG-TRDE	YGCGIYLSPDD	LENPREYIVWN
	RcSRO2b	VKYAWFGASSRDD	YGCGIYLSPDD	FLSPKKYIVWS
	VvSRO2b	VKFGWYSA-SKDE	YGCGVYLYPHH	LPAPKKYIVWS
	AlSRO2d	VKYGWCSV-AKQE	FGRGLYLSPDN	LASPKKYIVWS
	AtSRO5	VKYGWCSV-SKHE	FGRGLYLSPDN	LVSTKKYIVWS
	AlSRO2c	VKYGCCGV-EKEE	SHNGLCLSPDN	LISPKKYMIWS
	AtSRO4	VKYGCCGV-EKEE	SNNALCLSPDN	LTSPNKYIIWS
	AtPARP1	KMLLWHGS-RLTN	FGKGIYFADLV	ELMYNEYIVYD
	AtPARP2	RMLLWHGS-RLTN	FGKGVYFADMF	MLLYNEYIVYN
	AtPARP3	KVLLWCGS-RSSN	FGRAIVCSDAA	PLEYNEYAVYD
