# Supplementary material for: The RST and PARP-like domain containing SRO protein family: analysis of protein structure, function and conservation in land plants
Source: BMC Genomics. 2010 Mar 12;11:170. doi: 10.1186/1471-2164-11-170 (PMC2848248; doi:10.1186/1471-2164-11-170)
Supplement: Additional file 3 — Primers used for analysis. All primer sequences used for qPCR anaylsis in the manuscript are listed in this file. [file 1471-2164-11-170-S3.PDF]

Primers for cloning

SRO5 to pDEST32

SRO5_pENTR_F	CACCATGGATTACGTGAGAACCCAA
SRO5_pENTR_R	TTAGTGTTGTACTTTATGTCC

RCD1 and its truncated versions to pDEST32

RCD1_F	5'-GWF+TCATGGAAGCCAAGATCGTCAAG
RCD1_FL_R	5'-GWR+TTACAATCCACCTGCACCTTCTTC
GW_-10_R	5'-GWR+TTAGATGCTTCCTGGAATCTCCTT
GW_-21_R	5'-GWR+TTAGTTTTGAAGGGTTGTTATGGT
GW_-30_R	5'-GWR+TTATAGCAGATCATCTCCTACAAT
GW_-40_R	5'-GWR+TTATTTCCTGACAAACTCCGCTCT
GW_-50_R	5'-GWR+TTACTTATCCCTCAGTTGTTGGTA
GW_-60_R	5'-GWR+TTAGATCAACAACATGTCGTTCTC
GW_-70_R	5'-GWR+TTAATGTGAGATTGCTGCAAACAG
GW_-80_R	5'-GWR+TTATGGCATCCATGGAGATTTGGG
GW_-90_R	5'-GWR+TTAGCTTGAACCAACACTGTTTGC
GW_-100_R	5'-GWR+TTAACCTCTTGCTCCCTGGTTTGA
GW_-110_R	5'-GWR+TTAAGGAGGAAGATCCTTAGGTCC

SRO5 to pB7FWG2.0

SRO5_CDS_F	5'-GWF+ATGGATTACGTGAGAACCCAAG
SRO5_CDS_R2	5'-GWR+CGTGTTGTACTTTATGTCCACAAGC

RCD1 to pGEX4T-1

RCD1_pGEX_F	5'-GAATTCATGGAAGCCAAGATCGTCAA
RCD1_pGEX_R	5'-GCGGCCGCTTACAATCCACCTGCACCTTC

qPCR primers

RCD1_F	5’-GAGGTTCAGGAAGTGCAAACAGT
RCD1_R	5’-AACAGAGTAGGAAATGGCATCCA
SRO1_F	5’-TGGGTTAGATTAATGTGTCGACTTCT
SRO1_R	5’-CTAAGTTTCTAAAGATCAAGTCACGTTTG
SRO2_F	5’-CACGCCGGATCTTCTTTCTC
SRO2_R	5’-AACGTCGTGCTCTGGGTTTC
SRO3_F	5’-ATTTAGACAACGGCGAAATCATC

GWF = Gateway forward sequence
GGGGACAAGTTTGTACAAAAAAGCAGGCT

GWR = Gateway reverse sequence
GGGGACCACTTTGTACAAGAAAGCTGGGT

SRO3_R	5’-GTTGCATCGCCGGAGAAG
SRO4_F	5’-TTGTGATCAACAACATTCTTCCATAG
SRO4_R	5’-CGGTAGATAAGCTCGTAAACTTTATCG
SRO5_F	5’- TTACACGGAGCGAACTAATCCA
SRO5_R	5’- TGTCCACAAGCTTTGATGATGTG
Actin-2 _F	5’-ATCAATTCGATCACTCAGAGC
Actin-2 _R	5’-CTATGATGCACTTGTGTGTGA
At1g62422_F	5'-AGAGGCGGAGAAGGAGAAAG
At1g62422_R	5'-CCCCAAACTCCCAAAAATCT
At3g42860_F	5'-TGATGCGGTTCAGTCTTCTG
At3g42860_R	5'-CACACGGACACTGATGATCC
At5g14600_F	5'-ACCGTTCAAACGAAAACGAC
At5g14600_R	5'-CCATGACGACTGAAGAAGCA
At1g12970_F	5'-GGTTAACCAAAGCGCTGAAG
At1g12970_R	5'-TGCTCCTCCTTGTTGCTTCT
At3g30720_F	5'-TGAAAGGTTCATTTTGCCTCA
At3g30720_R	5'-GACCCTCATTTTGAGCCTTG
At3g47060_F	5'-TTCATTGATGAGCTGCTTGG
At3g47060_R	5'-TCAGTTGCCCGCCTTATATC
At1g50890_F	5'-TGTGGCTGGAGAAGACTGTG
At1g50890_R	5'-CCATCATTGCTTCTGCTTCA
At1g69680_F	5'-AGCTGTCCCAGCTTCACAAT
At1g69680_R	5'-CAAGCTGAGGAACCGAAAAG
At1g56720_F	5'-CATTTGGGTACGTTGCTCCT
At1g56720_R	5'-CGTAATCCACAGGGTCTCGT
At3g43290_F	5'-GTCGCAGAGAAGACCCTCAG
At3g43290_R	5'-GAGACATCCCTTGCAACCAT
At3g14300_F	5'-GACGGGGTTCGTACTTTCAA
At3g14300_R	5'-GCTACCGCTTGATGCTTCTC
At1g79410_F	5'-TTGGGTTGGGAATGTCGTAT
At1g79410_R	5'-AACTCCACCATGGCGTTTAG
